# Crosstalk Between Glycinergic and N-Methyl-D-Aspartate Receptor-Mediated Glutamatergic Transmission in Behaviours Associated with Opioid Use Disorder

**DOI:** 10.3390/ijms262110526

**Published:** 2025-10-29

**Authors:** Nariman Essmat, Imre Boldizsár, Yashar Chalabiani, Bence Tamás Varga, Sarah Kadhim Abbood, Judit Mária Kirchlechner-Farkas, Kornél Király, Ildikó Miklya, István Gyertyán, Tamás Tábi, Susanna Fürst, Laszlo G. Harsing, Ferenc Zádor, Mahmoud Al-Khrasani

**Affiliations:** 1 Department of Pharmacology and Pharmacotherapy, Faculty of Medicine, Semmelweis University, Nagyvárad tér 4, H-1085 Budapest, Hungary; nariman.gomaa@phd.semmelweis.hu (N.E.); boldizsar.imre2@semmelweis.hu (I.B.J.); chalabiani.yashar@semmelweis.hu (Y.C.); varga.bence@phd.semmelweis.hu (B.T.V.); abbood.sarah@phd.semmelweis.hu (S.K.A.); kirchlechner.farkas.jmi@gmail.com (J.M.K.-F.); kiraly.kornel@semmelweis.hu (K.K.); miklya.ildiko@semmelweis.hu (I.M.); gyertyan.istvan@semmelweis.hu (I.G.); furst.zsuzsanna@semmelweis.hu (S.F.); harsing.laszlo@semmelweis.hu (L.G.H.J.); zador.ferenc@pharma.semmelweis-univ.hu (F.Z.); 2 Center for Pharmacology and Drug Research & Development, Semmelweis University, Ülloi út 26., H-1085 Budapest, Hungary; tabi.tamas@semmelweis.hu; 3 Department of Pharmacology and Toxicology, Faculty of Pharmacy, Zagazig University, Zagazig 44519, Egypt; 4 Department of Pharmacodynamics, Semmelweis University, Nagyvárad tér 4, H-1089 Budapest, Hungary

**Keywords:** OUD, NMDAR, GlyT-1, GPR158, DA, GABA, reward system

## Abstract

The current pharmacological approach for the treatment of opioid use disorder (OUD), as a result of prescription misuse or illicit opioids, utilises opioid ligands that have either an agonist or antagonist profile. In this context, methadone and buprenorphine act as opioid agonists, whereas naltrexone functions as an opioid antagonist. To decrease the reinforcing effects of illicit opioids, higher doses of methadone and buprenorphine have been recommended, but this is associated with increased side effects. Therefore, several preclinical efforts have been carried out over the last decades to find drugs that act on receptors other than opioid receptors. A large body of preclinical evidence has shown the ability of N-methyl-D-aspartate receptor (NMDAR) antagonists like ketamine to treat opioid addiction behaviours in animals. Indeed, ketamine by itself is an addictive drug; thus, the treatment of OUD is still a matter to be solved. Growing data position glycine transporter 1 as a possible therapeutic target for the treatment of substance use disorder. This transporter regulates the reuptake of glycine, which can modulate the function of both NMDARs and GPR158, a metabotropic glycine receptor (mGlyR); thus, it is worth investigating in the management of OUD. To gain insight into the role of glycinergic transmission in OUD, alongside NMDAR-mediated glutamatergic transmission, dopaminergic and GABAergic transmission were also reviewed.

## 1. Introduction

The history of opioid addiction can be traced back to the beginning of the 16th century, where physical dependence was described as the appearance of withdrawal symptoms upon the cessation of opium use [1]. The breakthrough in opioid use began in the 19th century with the extraction of morphine, the first active constituent of opium, yet the invention of hypodermic needles facilitated its intravenous (IV) use. This has led to massive advancements in pain management; unfortunately, reports concerning the prevalence of opioid addiction and, consequently, overdose fatalities have emerged [2]. Next to the extraction of morphine, scientists developed a semisynthetic derivative in the hope of creating opioids that are devoid of addiction. However, instead of fulfilling this hope, opioid addiction further worsened, particularly with the introduction of new semisynthetic opioid analogues, specifically heroin, and later with the development of synthetic analogues such as fentanyl. Fentanyl and its analogues have been counted as illicit substances, distributed per se or as an adjuvant to heroin to increase its perceived potency [3], playing a major role in the recent rise in overdose-related fatalities [4]. At present, novel synthetic opioids (NSOs), which include both fentanyl analogues (fentalogs) and non-fentanyl opioids that interact with the µ-opioid receptors (MORs), are among the drug classes that contribute significantly to overdose-related deaths [5]. In addition to illicitly used opioids, prescribed opioid analgesics like oxycodone have also contributed to the increase in the incidence of opioid addiction and overdose deaths [6]. Several strategies have been followed, including medications of either an opioid or non-opioid-based nature, immunotherapies, as well as non-pharmacological approaches, which have been developed either to prevent or to treat opioid use disorder (OUD) [7,8,9,10]. OUD falls under the category of substance use disorders (SUD) and encompasses an intense craving for opioids, heightened opioid tolerance, and withdrawal symptoms upon discontinuation of opioid use [11,12].

Several pieces of evidence at the level of preclinical studies regarding withdrawal symptoms of OUD have shown the impaired function of certain brain areas of the physiological rewarding system associated with opioid agonist use. In this context, mice were implanted with a cannula positioned near the lateral hypothalamus (LH), medial hypothalamus (MH), periaqueductal gray (PAG), dorsal (DRF) or ventral parts (VRF) of the reticular formation, ventral tegmental area (VTA), above either the nucleus accumbens (NAc), the anterior caudate putamen, or the posterior caudate putamen and allowed to self-administer morphine via the cannula during a spatial discrimination task using the Y-maze test. Those animals that received a cannula in the proximity of the MH, PAG, VRF, and medio-ventral NAc showed reinforced behaviour in the Y-maze test [13,14].

Other studies have shown that bilateral microinjections of morphine into the VTA led to naloxone-sensitive alterations in conditioned place preference (CPP). Yet, systemically administered morphine-induced CPP was blocked by naloxone methiodide injected into the VTA and PAG [15,16]. Furthermore, bilateral infusion of MOR-specific siRNA into the substantia nigra (SN) and VTA attenuated heroin-induced increases in locomotor activity and CPP. This effect of MOR-specific siRNA is likely the result of a reduction in MOR mRNA and protein. These data point to the role of the NAc in reward-motivated action of animals induced by MORs activation.

The current opioid-based medications act on MORs either as agonists or antagonists, whereas the non-opioid-based drugs act on non-opioid receptors (non-ORs). Opioid-based approach, namely medications for opioid use disorder (MOUD), methadone, buprenorphine, and naltrexone are available. Methadone and buprenorphine are the most widely used medications for treating OUD in the USA and other countries. The evidence base supporting their use is stronger compared to that for naltrexone [8,9,10,17,18]. On the other hand, drugs that act on non-ORs have gained considerable attention in preclinical studies in the hope of developing new strategies for managing OUD. Among these receptors, dopamine (DA), N-methyl-D-aspartate (NMDA), and gamma-aminobutyric acid (GABA) receptor systems are involved in the modulation of OUD [19]. In this context, a growing body of experimental data indicates that the excessive activation of NMDA receptors (NMDARs) in the NAc plays a significant role in the development of OUD (opioid addiction and withdrawal). This is supported by evidence showing that the expression of the NMDAR subunit GLUN2B increased in the NAc and hippocampus (Hipp) in rats that exhibited morphine-induced CPP. At the same time, an NMDAR antagonist was found to dose-dependently reduce morphine-induced CPP [20,21,22].

These changes were not observed in the case of natural rewards such as food, novel environment, or social interaction. Western blot analysis revealed that the expression of GLUN2B increased in the NAc and Hipp in rats with morphine-induced CPP. In addition to this, GLUN2B-containing NMDAR inhibition in the Hipp and NAc shell (NAcSh) suppressed reinstatement of morphine CPP [23]. Furthermore, glutamate, α-amino-3-hydroxy-5-methyl-4-isoxazolepropionic acid (AMPA), and NMDA significantly increased GABA release from NAc core (NAcC) slices obtained from rats that were treated with a single systemic morphine dose three weeks prior [24]. This was associated with increased transcript levels of NMDAR and AMPA/kainate receptor subunits. These indices collectively underscore the essential role of NMDARs and the need for further investigation of drugs that modulate their function in OUD treatment. Glycine is a co-agonist of NMDARs, and changes in its levels result in changes in the function of the NMDARs. Recently, we reported on the ability of a glycine transporter 1 (GlyT-1) inhibitor to minimise the development of morphine analgesic tolerance [25,26].

This review highlights the implications of NAc DA, GABA, NMDARs, and, last but not least, GlyT-1 and GPR158, a metabotropic glycine receptor (mGlyR), in the development of OUD.

## 2. Opioid Use Disorder and Dopamine Hypofunction

Over the past decades, researchers have focused on the functional changes that occur in the VTA-NAc as a consequence of chronic opioid use for either medical or recreational purposes. In this regard, it has become evident that opioid agonists can indirectly influence DA in NAc, which is considered a crucial area concerning reward-related behaviour participating in the development of OUD [27,28]. Nevertheless, other brain areas, such as the nucleus of the amygdala and the stria terminalis, among others, are also involved in the development of OUD [13,14,29]. For a comprehensive review that provides additional details on the neuronal inputs and outputs of the NAc, refer to Baik 2020 [30].

Early evidence shows a dose-dependent increase in DA release in the rat NAc following systemic, VTA, or NAc fentanyl treatment [31]. In this study, the authors have shown the involvement of mesolimbic MORs and δ_2_-opioid receptor (DOR2) in the measured effect of fentanyl as indicated by the use of non-selective and subtype-selective MOR and DOR antagonists. This study, among others, has revealed the important physiological role of MOR and DOR types that can enhance DA in rats’ NAc. As mentioned above, this review summarises the role of the MORs in the development of OUD. In this section, we will focus on the impact of MOR agonist treatments on the DA signalling pathway, particularly concerning its increase and decrease in OUD.

Heroin is one of the most well-known opioid agonists that can induce OUD [32], and a preclinical study carried out by Wise and coworkers has proved that heroin self-administration in rats significantly increased DA and its metabolite 3,4-dihydroxyphenilacetic acid (DOPAC) levels (up to 300%) in NAc [33]. In addition, higher doses of heroin further increase DA levels in the NAc, and ceasing heroin administration results in the restoration of DA levels, thus indicating that heroin strongly activates the brain’s reward system [33].

A recent investigation focused on the responsiveness of DA release, triggered by both acute and chronic morphine administration, to naloxone treatment given either before or after morphine treatment in the medial or lateral shell of the mouse NAc [34]. In the morphine treated group, acute IV morphine injection has induced anatomically distinct DA increases in the medial and lateral NAc. The medial DA response to morphine was surprisingly sensitised by long-term morphine treatment, in contrast to the decreased response in saline-treated mice [34]. In addition, this study tested the impact of naloxone induced withdrawal on morphine induced DA release in NAc. The study showed that the injection of naloxone was effective in reversing the increased DA tone that occurred after morphine injection; this effect was sustained even after long-term morphine injection in mice [34].

It was also found that the acute effect of morphine and oxycodone on NAc DA level was abolished by pretreatment with naloxone [35]. Indeed, the work has also highlighted the difference in the effect of these opioid agonists on DA levels, namely, oxycodone induced a rapid and stable elevation in DA levels; however, morphine induced a brief elevation in DA levels in the NAc [35]. In a study designed to evaluate DA release in the NAc of rats subjected to chronic treatment with increasing doses of morphine, the authors have shown that chronic morphine increased accumbal DA release. The challenge morphine doses injected either 3 or 30 days after the last morphine injection elicited a significant augmented DA release compared to controls [36].

The effects of morphine on DA levels and its metabolites, specifically DOPAC and homovanillic acid (HVA), demonstrate both dose-dependent and biphasic patterns in the NAc and the striatum of rats, as has been documented in various studies [37,38].

In relation to the modulation of DA and its metabolites under conditions of reward induced by opioid use and aversive states associated with withdrawal, Pothos and co-workers found that the level of DA and its metabolites increases in NAc after acute and 7-day chronic systemic morphine treatments. In addition, systemic naloxone injected at day 8 precipitated withdrawal in rats associated with low DA levels in the NAc. On the other hand, systemic clonidine treatment in dependent rats reduced the withdrawal symptoms, such as wet teeth-chattering and dog shakes, as well as reduced DA release in rats’ NAc [39]. Another early study has shown that rats treated with increasing morphine doses for 15 days exhibited morphine dependency, indicated by the appearance of withdrawal symptoms after naloxone treatment, such as wet-dog shakes, jumping, and tooth chattering, among others. Regarding changes in DA homeostasis, one day after morphine withdrawal, morphine dependent rats showed much lower DA output (about 80%) compared to controls in the caudal NAc-ventral striatum. A challenge morphine dose in morphine-dependent rats elevated DA output only in the saline group (about 45%), with no change in morphine dependent rats one day after morphine withdrawal [40].

In another study, rats were trained to self-administer oxycodone, after which an addiction test was conducted with a lower concentration solution. Following the experiment, withdrawal was induced, with one group terminated after 1 day and another after 2 weeks. DArgic activity in the NAc was examined using ex vivo brain slices/synaptosomes instead of microdialysis. While electrically stimulated DA release remained unchanged, DA reuptake decreased. Additionally, DA reuptake was reduced in naïve animal samples treated with oxycodone and [D-Ala^2^, MePhe^4^, Gly(ol)^5^] enkephalin (DAMGO). Notably, after two weeks (but not after one day), phosphorylation of the DA transporter (DAT) decreased, even as uptake levels remained consistent across both time points. The article’s methodology seems to overlook analysing changes in NAc input rather than just local DA release. During the forced withdrawal following dependence, the electrically stimulated DA release in isolated NAc did not change; however, reduced DA uptake was measured in the tissue. Acute opioid treatment also decreased DA reuptake in tissues isolated from naïve animals [41].

The above-mentioned studies and data in Table 1 support that chronic opioid exposure creates significant alterations in DA neurotransmission, manifested by an increase in DA level changes within the NAc. These changes constitute both rewarding and aversive behaviour states associated with OUD. The complex interactions between opioid and DArgic systems and the effects on DA levels remain a captivating subject for ongoing research, particularly in the context of understanding OUD and identifying potential therapeutic targets. Therefore, our review in the next sections will focus on the possible indirect effects of MOR on NAc DA, as well as the interplay between these neurons, GABAergic neurons, and the potential impact of NMDARs when glycine levels are altered in response to the inhibition of GlyT-1.

## 3. The Interaction Between Opioids and the GABAergic System in Response to Exposure to Mu-Opioid Agonists

The essential function of GABAergic neurons in OUD is integral to their significant impact on maintaining the excitation/inhibition equilibrium within the mesolimbic DArgic system, which serves as the major centre (pathway) for both reward and aversive behaviours [30,43]. In the NAc, two GABAergic medium spiny neuron (MSN) populations are distinguished, comprising 90–95% of the total, characterised by the expression of D1 receptors (D1-MSNs) and D2 receptors (D2-MSNs); however, they show different distribution patterns [30,44].

The colocalization of MORs and GABARs on the same neurons, as well as the presence of MORs on GABAergic neurons, highlight the interplay between these two systems that manifests in dysregulation of the GABAergic system in OUD [45]. A review article by Hosseinzadeh Sahafi et al. [46] highlights that ORs are frequently co-expressed on GABAergic interneurons and modulate GABA transmission, often by reducing pre-synaptic GABA release or hyperpolarizing interneurons, leading to disinhibition of excitatory outputs.

**Figure 1:**
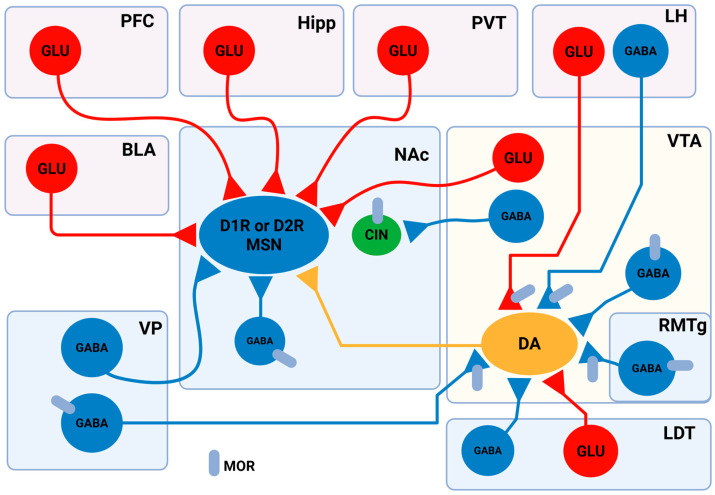
Schematic and simplified representation of the neural connections between brain regions involved in the reward system. In the NAc, neurons are categorised into three main types: MSNs, local GABAergic interneurons, and cholinergic interneurons (CINs). MSNs express either DA D1 receptors (D1R) or D2 receptors (D2R), and are classified as D1R-MSNs or D2R-MSNs, though the percentage of these cells in the NAc region varies, with 13.73–17% of NAcSh cells and 5–14.05% of NAcC cells co-expressing these receptors. MSNs receive glutamatergic inputs from several brain regions, including the PFC, basolateral amygdala (BLA), Hipp, the nuclei of the paraventricular thalamus, and the VTA. MSNs are also innervated by local GABAergic interneurons and long-projecting GABAergic neurons located in the ventral pallidum (VP). Importantly, MSNs receive DArgic innervation from VTA DArgic neurons. Besides MSNs, CINs also express DRs. CINs are innervated by GABAergic input from VTA neurons, which preferentially target CINs in the NAc. In the VTA, DAergic neurons are under the influence of local GABAergic interneurons. The rostromedial tegmental nucleus (RMTg), the lateral dorsomedial tegmentum (LDT), and the VP make GABAergic synapses with the VTA DAergic neurons as well. Glutamatergic LDT neurons also send projections to VTA DAergic neurons. A subset of aversion-linked VTA DA neurons is controlled by glutamatergic input from the LH, which also provides GABAergic input to reward-related VTA DA neurons. Several neurons express MORs in the reward system, including local GABAergic interneurons in the VTA, GABAergic RMTg, and VP projecting neurons. MORs are also expressed on VTA projecting aversive stimuli linked to LH neurons. In the NAc, CINs are MOR-positive cells. Adopted from [80,82,83,100,101,102,103,104,105,106,107,108,109,110,111]. Created in BioRender. Gergely, T. (2025) https://BioRender.com/3zdhnmx.

The likelihood of elevated GABA release during withdrawal in brain slices from animals that had been treated chronically with morphine [47] points to the role of GABA in OUD. This supports the view that opioids such as heroin, morphine, and fentanyl initially exert reinforcing effects by suppressing GABAergic interneurons in the VTA, thereby disinhibiting DA neurons and enhancing mesolimbic DA release Figure 1 [48,49]. The opioid antagonist sensitivity of MOR-selective agonist DAMGO, which decreased the activity of rostromedial tegmental (RMTg) neurons, while suppressing large-amplitude spontaneous GABA-A inhibitory postsynaptic currents (IPSCs) in VTA DA neurons, supports the interaction between opioid and GABAergic systems. The activation of RMTg neurons caused GABA-A IPSCs in DA neurons. These GABA-A IPSCs were decreased by the MOR activation. According to these results, RMTg sends opioid-sensitive input to DA neurons in the midbrain [49]. Based on a review carried out by Xi and Stein, GABA_A_ or GABA_B_ agonists γ-vinyl GABA (GVG) and baclofen are capable of decreasing heroin-induced DA release in VTA and reducing substance abuse behaviour, indicating the implication of GABAergic modulation in opioid reinforcement [48]. In a rodent model of morphine withdrawal, it was found that withdrawal significantly enhanced glutamatergic excitation and suppressed GABAergic inhibition in amygdaloid central nucleus (CeA) projection neurons, leading to increased neuronal firing. Glutamatergic inputs from the parabrachial nucleus to the CeA further exacerbated CeA hyperactivity and withdrawal behaviour, whereas stimulating local GABAergic interneurons reduced both neural excitability and somatic symptoms. These findings also indicate that opioid withdrawal disrupts inhibitory control in the CeA, causing disinhibition of excitatory input and hyperactivation of CeA output neurons, which together mediate the symptoms of withdrawal. The outcome suggests that restoring GABAergic tone or suppressing excessive glutamatergic input in the CeA could be a valuable strategy to reduce withdrawal severity [50]. In another study conducted on rats that underwent morphine withdrawal, it was revealed that the activity of CeA GABAergic neurons increased following acute morphine administration, and this morphine-induced excitation of inhibitory neurons was protein kinase A (PKA) dependent [51].

Attempts to further support the changes in the levels of GABA under opioid use include the ability of a single intranasal buprenorphine administration to evoke an increase in GABA and a decrease in DA levels in the brains of rats. These results suggest that buprenorphine counteracts opioid-induced neurotransmitter dysregulation by increasing GABA and decreasing DA, likely reducing stimulant and reward signalling associated with addiction. Simultaneous brain-derived neurotrophic factor (BDNF) upregulation—possibly cyclic AMP response element-binding protein (CREB)-mediated—may enhance neuroplasticity and recovery from opioid-induced neuroadaptations [52]. In a human study, it was shown that patients with prescription opioid addiction had significantly lower GABA and higher glutamate levels than controls. With the help of the Barratt Impulsiveness Scale and the Montreal Cognitive Assessment (MoCA) tests, it was determined that lower GABA predicted higher impulsivity and poorer cognition. Conversely, glutamate levels showed a positive correlation with impulsivity but no significant association with MoCA scores. Mechanistically, the study suggests that prescription opioid addiction is associated with an imbalance in excitatory/inhibitory neurotransmission in the prefrontal cortex (PFC), with reduced GABAergic inhibition and elevated glutamatergic excitation contributing to impulsive behaviour and cognitive impairment [53]. In mice with a specific gene variation known as the human single-nucleotide polymorphism (SNP) (A118G), which is linked to a higher risk of addiction, a corresponding knock-in mouse model carrying a similar mutation (A112G) was used to explore this connection in the effect of morphine. In normal (wild-type) mice, morphine-mediated MOR activation increases the firing rate of DArgic neurons located in the medial nucleus accumbens shell (mAcbSh). This highlights the suppression of DArgic activity as a consequence of a reduction in the activity of local GABAergic inhibitory neurons. However, in the mutant mice, both of these effects are less pronounced. Additionally, the increase in movement (locomotor activity) that typically results from MOR activation is also diminished in the mutant mice [54].

In a rodent model of oxycodone withdrawal, long-lasting anxiety-like behaviour developed, accompanied by a significant downregulation of GABA_A_ receptor α2 and γ2 subunits and upregulation of GABA_A_ α1 and β2 in the CeA of rats that accessed long IV oxycodone self-administration. The notion is that extended oxycodone intake induces lasting reconfiguration of GABA_A_ receptors in the CeA, which may contribute to the emergence of negative affect and stress sensitivity that underlie relapse vulnerability in OUD [55]. A retrospective clinical study by Hermenau and his coworkers compared the clinical effectiveness of the partial MOR agonist buprenorphine with the GABA_B_ receptor agonist baclofen for managing acute opioid withdrawal. It was found that the primary outcome, detoxification success (defined as no need for additional ‘as-needed’ (PRN) withdrawal medications), was significantly higher in the baclofen group compared to the buprenorphine group [56].

More recently, it was shown that spontaneous heroin withdrawal-induced hyperalgesia is associated with activation of dorsal raphe (DR) neurons expressing MOR mRNA, particularly those co-expressing vesicular GABA transporter (VGaT), vesicular glutamate transporter 3 (VGluT3), and tryptophan hydroxylase (TPH), but not those expressing TPH alone. Selective inhibition of DR-VGaT (GABAergic) neurons significantly attenuated hyperalgesia during withdrawal. The mechanism proposed is that during withdrawal, activation of MOR-expressing DR-GABAergic neurons increases GABA release. These GABAergic neurons inhibit local serotonergic neurons, reducing serotonin output, thereby enhancing pain sensitivity. Additionally, the activation of MOR-VGluT3 and MOR-VGluT3-TPH neurons may contribute to the excitatory drive [57].

A recent postmortem study carried out by von Gilsa et al. (2025) on brain tissues from patients who died from heroin overdose vs. those who suddenly died from natural causes aimed at investigating if long-term heroin use alters the GABAergic system in the anterior midcingulate cortex (aMCC) in layers III and V [58]. Glutamate decarboxylase (GAD)65/67-immunoreactive neuropil density was significantly reduced in layer V of the left aMCC in heroin users, with no significant bilateral increase in the density of GAD-positive somata. This suggests presynaptic terminal loss or decreased GABA production, which may contribute to disinhibition of pyramidal output neurons in layer V of the aMCC, alongside increased aMCC activity seen in neuroimaging studies of opioid craving. In contrast, the same group found significantly elevated neuropil GAD65/67 immunoreactivity in layer V in the anterior insular cortex (AIC) in the same postmortem samples [59]. Whereas the observed decreased GABAergic inhibition of the cortical output neurons in the aMCC aligns with the efficacy of GABAergic agonists in experimental models of OUD [60,61,62,63], the opposite change in the AIC highlights that different cortical regions may be part of different neuronal networks involved in and modulating the complex effects of chronic opioid intake.

Several studies show the ability of GABA receptor positive allosteric modulators (PAMs) such as KK-92A and ASP8062 to attenuate opioid-seeking and relapse in rodent and primate models, without the sedative effects induced by full agonists [60,61]. For example, the GABA_B_-receptor PAM such as KK-92A significantly inhibited both cue- and drug-induced reinstatement of fentanyl seeking in rats without affecting sucrose-seeking, suggesting specificity for drug-related behaviours. In TH+ VTA DArgic neurons, the mRNA of G-protein-gated inwardly rectifying potassium channel (GIRK) 2 and 3 significantly decreased, while GABA_B_ R1 and regulator of G protein signalling 2 (RGS2) levels remained unchanged [60]. Likewise, another PAM, ASP8062, significantly reduced morphine intake in nonhuman primates. The authors predicted that ASP8062’s action involves GABA_B_-mediated inhibition of DArgic transmission in the mesolimbic pathway, reducing morphine’s reinforcing properties without the sedative or respiratory side effects seen with GABA_B_ agonists like baclofen [61]. The interaction between the GABAergic and MOR systems is outlined in Table 2.

In the central nervous system (CNS), GABAergic interneurons could be categorised based on the expression of different markers such as parvalbumin (PV), somatostatin (SST), vasoactive intestinal peptide (VIP), and calretinin [64]. MORs and PV-interneurons exhibit significant colocalization within corticolimbic regions like hippocampal CA1, suggesting a functional relationship between them [65]. MORs are significantly localised at GABAergic presynaptic terminals associated with interneurons, contributing to a disinhibitory effect on hippocampal pyramidal cells. In this regard, MOR activation inhibits GABA release from the terminals of the PV-expressing interneurons onto the CA1 pyramidal neurons [66]. These studies support that MOR activation results in disinhibitory network effect within the Hipp and is primarily associated with PV-expressing interneurons [65,66]. Moreover, a recent study by Caccavano has shown that opioids are selectively positioned to inhibit hippocampal PV-expressing interneurons while exerting minimal effects on their neocortical counterparts [67]. Nevertheless, this difference is highly conserved across various species, including mice, macaques, and humans. SST-expressing interneurons, another type of GABAergic interneuron, are distributed within areas involved in the regulation of the reward system, such as the Hipp and VTA. In VTA, these interneurons, along with other interneurons, regulate dopaminergic activity and reward behaviours, increasing dopamine release when MORs are activated [46,68,69]; see Table 2. Within the hippocampal CA1 region, these interneurons also express MORs, and their activation produces a similar effect to that observed with the activation of MORs in PV-interneurons in the same region [67]. Indeed, in the CA1 layer of the Hipp, SST or neuropeptide Y are expressed in oriens-lacunosum moleculare (O-LM) interneurons, which are GABAergic interneurons innervating the distal dendrites of pyramidal cells [70]. Understanding the distribution and function of MORs in SST-expressing interneurons broadens our knowledge of their contributions to hippocampal functions, particularly in the context of learning and memory, and underscores the importance of interneuron diversity in regulating neuronal circuits. In the striatum, the stimulation of these interneurons results in a simultaneous excitatory and inhibitory response through the co-release of GABA and glutamate onto various postsynaptic targets [71]. Overall, the integration of SST in the signalling pathways related to opioid release and the resultant effects on VTA and hippocampal neural activity underscore its crucial position in the reward system and pain perception, suggesting that SST-expressing interneurons are fundamental contributors to these processes [46,72]. It has been recognised that VIP is expressed in a significant subpopulation of inhibitory interneurons, particularly in the Hipp [73], forming inhibitory synapses with other interneurons that ultimately result in disinhibition [74]. In addition, the hippocampal CA1 region contains cells that express met-enkephalin, which co-localises with VIP and calretinin [75]. Furthermore, morphine intake voluntarily strengthens mRNA levels of opioid receptors in the PFC but does not change VIP levels; however, the result in the NAc was contradictory, with a slight elevation in mRNA levels of VIP in morphine-administered rats [76]. Research indicates that a balance in VIP interneuron activity is essential for spatial learning, which is negatively affected by OUD [77]. In addition to the above-mentioned VTA GABA neurons, they also express calretinin alongside the amygdala, and PFC–hippocampal reward circuits [78,79]. Calretinin appears to significantly influence opioid receptor responsiveness and dopamine release via intricate calcium signalling mechanisms in GABAergic interneurons. This understanding is crucial for comprehending the broader implications of opioid signalling in neural circuits, which can have considerable ramifications in the context of addiction and pain management [46]. Currently, there is no known molecular or physiological-based evidence that can specifically distinguish VTA GABA interneurons from GABA projection neurons [68,80]. Thus, identifying a unique marker for VTA interneurons would facilitate research into their specific roles compared to projection neurons. On the other hand, the shared mechanisms between the opioidergic and GABAergic systems across different corticolimbic regions indicate that therapeutic strategies should consider how these systems interact in OUD alongside other mood-related disorders. Furthermore, evidence has demonstrated that morphine caused varying degrees of inhibition in RMTg, NAc, and VTA GABAergic neurons [81]. Based on this finding, it could be assumed that MOR agonists may activate MOR to a varying extent in different reward behaviour-regulating CNS regions. Further studies are needed to determine whether MOR agonists preferentially activate MOR in specific subtypes of GABAergic interneurons. GABAergic interneurons and GABAergic neurons in general are abundant in several CNS regions, which are responsible for the manifestation of different opioid effects. GABAergic interneurons are located in several regions of the CNS, including the reward system, such as the NAc, VTA, and RMTg, as well as the respiratory system, PAG, and spinal dorsal horn [82,83,84,85,86,87,88,89]. It is worth noting that in the spinal cord, GABAergic interneurons express PV and nitric oxide synthase, but not calretinin or somatostatin, which are expressed mainly in excitatory neurons [90,91,92]. Another factor to consider is the possible variants of MOR expressed across the different regions. Numerous MOR splice variants have been identified. The regional distribution of these MOR subtypes is varied. For example, in humans, MOR-1G2 mRNA is highly expressed in several CNS regions, including the NAc and pons, but not the spinal cord. In mice, the expression of mMOR-1E mRNA is high in the striatum, whereas mMOR-1D mRNA level is higher in the brainstem and the PAG. These findings indicate that different regions, responsible for the manifestation of different opioid effects, express different MOR mRNA splice variants. However, it should be taken into consideration that on the protein level, other MOR variants were detected. Interpreting MOR protein variant distribution is challenging due to combined 3′ and 5′ splicing and the limitations of immunohistochemistry [93]. It was also found that 6TM MOR variants are important for the analgesic effects of a particular MOR agonist [94]. Indeed, an in-depth investigation of the distribution of the specific MOR variants in different GABAergic neurons is needed to determine the expression pattern of MOR splice variants in GABAergic neurons in different CNS regions. For a comprehensive understanding of the interaction between GABA interneurons expressing PV, SST, VIP, or calretinin in corticolimbic reward systems, refer to cited reviews [46,80].

In summary, OUD involves complex, region-specific dysregulation of GABAergic neurotransmission that underlies many of its core behavioural and physiological manifestations. These findings highlight the therapeutic potential of targeting GABAergic circuits and receptors to restore inhibitory balance, improve affective regulation, and reduce relapse risk.

**Table 2:** Evidence on the interaction between MORs and GABARs is a critical component in the development and maintenance of OUD.

Opioid Ligand and Route of Administration	Subject	Findings	Reference
Fentanyl self-administration, IV, in rats Fentanyl, vapor self-administration in mice	Male and female adult Long Evans rats Male and female adult aged C57BL/6 mice	GABAB PAM (KK-92A) suppressed relapse in the VTA by reducing GIRK2/3 expression in DA neurons mediated by GABAB-R.	[60]
Buprenorphine intranasal administration	Male Sprague Dawley rats	Reversal of neurotransmitter dysregulation (↑ GABA/BDNF, ↓ DA) in the brain through BDNF/CREB modulation.	[52]
Fentanyl vapor self-administration Naloxone, IP	Male and female adult C57BL/6 mice	A reduction in GABAB receptor-mediated inhibition in VTA dopamine neurons during withdrawal.	[50]
DAMGO and morphine (bath perfusion in brain slice electrophysiology experiments)	Oprm1 A112G knockin mice	↓ MOR suppression of GABA and Glu release onto the mesolimbic VTA dopaminergic neurons (SNP effect).	[54]
Morphine pellet, SC Naloxone, SC	Male Sprague Dawley rats	Acute morphine application produces mixed effects on GABAergic transmission mediated by a switch in MOR G protein coupling to stimulatory Gs proteins, causing activation of the cAMP-PKA pathway that increases GABA release. Increased basal GABAergic transmission and mIPSC frequency in the CeA during withdrawal.	[51]
Daily morphine in neonatal rats, IP, and pellet implantations in adult rats, SC DAMGO–deltorphin for brainstem slices	Male Wistar rats	↓ GABA via DOR trafficking enhanced analgesia mediated by PLA2–AA–12-LOX–dependent inhibition of Ca^2+^ channels in the NRM.	[95]
Daily morphine in neonatal rats, IP, and pellet implantations in adult rats, SC. DAMGO–deltorphin mixture for isobologram	Male Wistar rats	A synergistic DOR–MOR interaction induced IPSC inhibition (PLA2 and cAMP/PKA dependent). In vivo, NRM microinjection of DOR and MOR agonists produced synergistic antinociception (PLA2-dependent).	[96]
Agonist, partial agonist and antagonist treatments for opioid addiction	Review	Opioids suppress GABAergic inhibition in the VTA, causing disinhibition of NAcC DA neurons, which enhances cue salience and increases relapse risk.	[97]
Increased morphine injection (10–20 mg/kg over 5 days, SC + 80 Hz whole body vibration)	Male Wistar rats	Reduced withdrawal symptoms associated with restored VTA GABA, increased DA release, and modulated expression of DORs on NAc cholinergic interneurons during withdrawal.	[98]
Increased heroin injection (5–40 mg/kg, twice daily, for 4 days, SC)	Male and female C57/B6 mice	Hyperalgesia was attenuated by DR GABA inhibition of serotonin and specific chemogenetic inhibition of DR-VGaT neurons.	[57]
Oxycodone self-administration, IV	Male HS rats	Highly addicted rats showed increased sIPSCs. Nociceptin reduces the excessive oxycodone self-administration and reverses GABA dysregulation in CeA.	[55]
Morphine (In vitro study)	Rat PAG slices	Opioid ↓ GABAergic synaptic currents in PAG via activating K^+^ channels through PLA2–AA–12-LOX pathway.	[87]
Morphine self-administration, IV or 10 mg/kg, SC (Respiratory depression)	Adult rhesus monkeys	ASP8062 decreased morphine self-administration without affecting the respiratory system via positive allosteric modulation of GABAB receptors (brain area was not specified).	[61]
Heroin overdose	Humans a postmortem case–control human study on 13 heroin-addicted males who died of overdose and 12 age-matched male controls who died of sudden natural causes	Decreased GAD 65/67-immunostained neuropil density in layers III and V, with a non-significant increase in somata density, indicating dysregulation of GABAergic interneuron-mediated inhibition in aMCC.	[58]
Heroin overdose	Humans 13 heroin-addicted males who died of overdose and 12 male controls who died of sudden natural causes	Bilaterally increased GAD65/67-immunoreactive neuropil density in layer V, which indicates dysregulation of GABAergic interneuron activity in AIC.	[59]
Codeine-containing cough syrup dependence	Human	↓ GABA+ and ↑ Glu levels in the medial PFC, where GABA+ levels correlated negatively with impulsivity and positively with cognitive performance; glutamate correlated positively with impulsivity but not with cognitive performance.	[53]
Fentanyl (48%) and other opioids (ie, morphine, hydrocodone, oxycodone, and hydromophone [48.6%]) as the primary substance of abuse	Human	Baclofen was more effective than buprenorphine in detoxification, and the effect was mediated via the VTA GABAB receptor.	[56]
OUD patients (Heroin)	Human	Hypermethylation in the promoter region of the *GAD2* gene. The peripheral blood samples were associated with OUD.	[99]

NRM: (Nucleus raphe magnus), PLA2: (Phospholipase A(2)), PKC: (Protein kinase C), N/OFQ: (Nociceptin/orphanin FQ peptide); ↑: increase; ↓: decrease.

## 4. Crosstalk Between N-Methyl-D-Aspartate Receptors and µ-Opioid Receptors in the Context of Opioid Use Disorder

Opioid use disorder induces profound, circuit-specific alterations in glutamatergic neurotransmission that encompass presynaptic release regulation, postsynaptic receptor remodelling, ion channel modulation, transporter dysregulation, and astrocytic and epigenetic control. Opioid receptors are expressed on glutamatergic projection neurons, interneurons, and astrocytes, where their activation modulates excitatory drive with remarkable pathway specificity. The focus of this section is on the interplay between NMDARs and MORs in the region that is largely involved in the aetiology of OUD. In the claustrum–anterior cingulate cortex (CLA–ACC) circuit, MORs and DORs selectively dampen polysynaptic recurrent excitation, whereas κ-opioid receptor (KOR) activation potently suppresses monosynaptic glutamate release presynaptically [112]. In the VTA, MORs are also expressed on a subset of VGlut2+ glutamatergic terminals that form monosynaptic excitatory inputs to DA neurons; MOR activation presynaptically inhibits these excitatory postsynaptic currents (EPSCs), gating NAc DA release and influencing self-administration and reinstatement [113,114]. While the classical model of opioid action in the VTA has focused on MOR-mediated inhibition of local GABAergic interneurons to disinhibit DA neurons, thereby promoting reward, the new data demonstrate a distinct, parallel mechanism involving MOR-expressing glutamatergic (VGlut2+) neurons, found primarily in the anterior VTA (Figure 1). These findings necessitate a revision of the canonical disinhibition model, incorporating the presynaptic MOR-mediated control of VTA glutamatergic neurons as a key regulator of mesolimbic DA signalling, opioid reward, and sex-dependent relapse vulnerability. This expanded understanding opens new avenues for targeting glutamatergic circuits in the VTA for the treatment and prevention of opioid addiction and relapses [114].

Chronic opioid exposure reshapes postsynaptic glutamate receptor composition and plasticity. In VTA DA neurons, a single morphine dose drives rapid insertion of GluA2-lacking, Ca^2+^-permeable AMPA receptors (AMPARs) through D1 receptor-dependent mechanisms [115]. With repeated exposure, multiple regions, including NAc, VTA, Hipp, show increased GluA1–3 and GluN2A/B expression, altered AMPA/NMDA ratios, and impaired long-term depression/potentiation [45,116,117]. Opposing adaptations in glutamatergic signalling underlie different stages of OUD. Acute opioids inhibit the release of glutamate, chronic use/withdrawal alters receptor expression and signalling pathways in NAc, VTA [45]. Distinct glutamatergic inputs to the NAc undergo unique projection-specific plasticity, which reflects the multifaceted role of the glutamatergic system in OUD, influencing both motivational states associated with drug use and the cognitive aspects of memory related to opioid experiences [116].

In Hipp, incubation of oxycodone craving is associated with persistent upregulation of AMPAR/NMDAR subunits and downregulation of presynaptic mGluR2/3, molecular changes that correlate with drug-seeking intensity [117]. Several glutamatergic circuits mediate incubation, relapse, and negative affect. Orbitofrontal cortex (OFC) → dorsal striatum (DS) glutamatergic projections with dorsal striatal D1R signalling drive incubation of oxycodone craving; disconnection or D1R blockade reduces seeking without affecting natural reward behaviour [118]. The circuit and receptor mechanisms underlying the incubation of oxycodone craving during abstinence were investigated, with particular focus on the glutamatergic projections from the OFC to the DS and D1R signalling in male rats. Activation of glutamatergic projections from the OFC to DS, along with concurrent D1R signaling in DS, is both critical for the time-dependent intensification (“incubation”) of oxycodone craving after abstinence. Coordinated OFC–DS circuit activity is required for the expression of incubated drug seeking, providing evidence for a circuit-level substrate underlying opioid relapse risk and identifying the OFC–DS glutamate/D1R axis as a promising target for intervention in OUD [118]. The role of glutamatergic neurons in the ventral pallidum (VPGlu) and their projection to the lateral habenula (LHb) in modulating heroin addiction and relapse was also explored in male rats [119]. The outcomes of this study pointed to a decreased VPGlu–LHb activity underlying heroin seeking, while restoration of VPGlu–LHb excitatory drive after extinction constrains relapse propensity, with the excitatory/inhibitory balance of ventral pallidal output to LHb critically determining motivation for opioid seeking. The study thus identified the VPGlu–LHb circuit as a pivotal substrate for context-dependent heroin relapse and a promising cell- and pathway-specific target for opioid addiction intervention [119]. Ventral pallidum glutamatergic outputs to lateral habenula suppress heroin relapse when activated, with extinction increasing excitatory marker VGlut2 and shifting the excitatory/inhibitory balance toward excitation [119].

In polysubstance paradigms modelling sequential clinical use of oxycodone and cocaine, it has been observed that oxycodone withdrawal is decreased with cocaine use. One explanation for this phenomenon is that synaptic depotentiation occurs in the NAcC, as indicated by a reduced AMPA/NMDA ratio and downregulation of glutamate transporter-1 (GLT-1) [120]. In the context of animal phenotype, a preclinical experimental study intended to dissect how sex and chronic immobilisation stress (CIS) affect glutamate receptor subunit trafficking in the hippocampal CA3 pyramidal neurons following oxycodone CPP, with a particular focus on NMDA (GluN1) and AMPA (GluA1) receptors. The study highlights the crucial interplay between sex, stress, and glutamatergic signalling in the Hipp during opioid addiction-related behaviours and provides a molecular explanation for observed sex differences in opioid addiction vulnerability, cue learning, and relapse risk [121]. In this regard, the hippocampal CA3 region and its glutamate receptor trafficking emerge as pivotal substrates mediating these phenomena, especially in the context of chronic stress and opioid exposure [121]. Furthermore, the study demonstrated that females had higher baseline GluN1 levels in CA3 dendrites than males, while baseline GluA1 levels in this location were similar in both sexes. Treatment with oxycodone significantly reduced GluN1 density and led to the redistribution of GluA1 from the membrane to the cytoplasm in females, decreasing its availability, which effects were not detected in males. CIS led to the redistribution of GluN1 in the CA3 region and decreased the availability of GluN1 in both sexes, albeit through different cellular mechanisms in each sex. Additionally, it increased GluA1 in males in small dendrites and mossy fibre synapses, a finding not observed in females. Administration of oxycodone after CIS also yielded sex-specific effects. In males, who did not develop oxycodone CPP, overall GluN1 density increased and so did GluA1 in the cytoplasm of CA3 dendrites and in the synapses of stratum radiatum spines, implying an enhanced glutamate binding capacity; in females, who developed oxycodone CPP, distribution of GluN1 remained similar to that of the saline treated CIS female group and GluA1 increased on the membrane of CA3 dendrites [121].

The activation of ionotropic or metabotropic glutamate receptors increased the activity of DArgic neurons in the VTA and induced the release of DA in the NAc and PFC, which enhances reward and facilitates heroin reinforcement in rats [122]. In addition, MOR activation modulates glutamatergic synaptic transmission at the parallel fibre–Purkinje cell (PF–PC) synapse in the mouse cerebellar cortex. MORs are functionally expressed on presynaptic glutamatergic axon terminals, where their activation suppresses glutamate release through G-protein–mediated inhibition of adenylyl cyclase and PKA activity, and possibly through β-arrestin–mediated extracellular signal-regulated kinases (ERK) phosphorylation. Although glutamate receptor subtypes or transporters were not directly examined, compelling evidence was provided that opioid exposure inhibits glutamatergic neurotransmission at the neuronal level by suppressing presynaptic release mechanisms, a process that may contribute to cerebellar dysregulation in the cognitive, affective, and reward-related sequelae of OUD [123]. Activation of ERK has also been recognised after morphine and cocaine treatments [124,125] in the reward and pain pathways. Furthermore, the activation of extrasynaptic NMDARs containing the GluN2B subunit and synaptic NMDARs shows a discrepancy in their impact on ERK; namely, the former inactivates, and the latter activates it, respectively [126]. For more details related to the implications of the ERK in SUD, refer to the work by Sun and colleagues [127]. A growing body of experimental data indicates that the excessive activation of NMDARs in the NAc plays a significant role in the development of opioid addiction and withdrawal. It was found that the expression of GluN2B increased in the NAc and Hipp in rats with morphine-induced CPP [22]. It was also shown that heroin relapse requires long-term potentiation (LTP)-like increases in the synaptic strength in the PFC projection to the NAc. Dendritic spine enlargement in accumbens spiny neurons was also observed, and an increase in the surface expression of GluN2B was also detected. The blockade of GluN2B before reinstating heroin-seeking prevented the induction of LTP-like changes in dendrite-remodelling and synaptic strength. The blockade of GluN2B also inhibited heroin relapse [128]. When an NMDAR antagonist or a D3R antagonist was co-administered with morphine, hyperlocomotion and behavioural locomotor sensitisation were significantly decreased. In morphine-treated animals, it was detected that the phosphorylation of GluN2B and total expression of GluN2B increased in the NAc. Systemic D3R antagonist reversed the morphine-induced changes in pGluN2B and GluN2B levels [129]. In rats, when a GluN2B-specific siRNA was directly injected into the NAc, chronic morphine treatment-induced CPP was suppressed but not morphine-induced behavioural sensitization [130]. It was also observed during acute morphine withdrawal that the phosphorylation of Ser897 in the GluN1 subunit (pGluN1) increased in the NAc. In neuronal cell culture, the pGluN1/GluN1 ratio and the surface expression of GluN1 increased. The increased pGluN1 level and the surface expression of GluN1 were dependent on PKA and NMDAR activity [131].

A translational study integrated human postmortem brain transcriptomics with machine learning and experimental rat models to identify molecular drivers of OUD, focusing on the glutamatergic system and its auxiliary proteins in the OFC. The study concluded that reduced Shisa7, an auxiliary protein of GABA_A_ and AMPA receptors that modulates their function and channel kinetics, is a marker of chronic heroin exposure. However, elevated OFC Shisa7 potentiates heroin seeking and impairs reward-related cognitive flexibility, supporting its role as a mechanistic driver and translational target in OUD-related glutamatergic and GABAergic dysfunction [132]. Shisa7 speeds up GABA channel deactivation and influences its trafficking, and increases AMPAR desensitisation and delays recovery from desensitisation [133,134]. It is expressed at higher levels across cortical regions and shows lower levels in subcortical regions in human and rodent brains [132].

An original human postmortem transcriptomic study examined whether molecular (transcriptional) rhythms are altered in the brains of individuals with OUD, focusing on two key brain regions implicated in addiction: the dorsolateral prefrontal cortex (DLPFC) and NAc. Chronic opioid use causes profound disruption in the temporal architecture of gene expression in corticostriatal circuits, altering the molecular rhythms governing glutamatergic, GABAergic, and DArgic neurotransmission [135].

The structural and functional plasticity of MSNs in the NAc in the context of OUD was elucidated in a detailed review of experimental and translational studies, with a special focus on glutamatergic synaptic mechanisms. This integrative review emphasises that targeting the synaptic and circuit-level adaptations of the glutamatergic system in the NAc may represent a promising avenue for novel OUD therapies [136]. The synaptic adaptations within cortico-accumbens glutamatergic circuits that drive OUD were discussed in a comprehensive review, focusing on preclinical rodent models and their translational relevance for relapse mechanisms. The review details how repeated opioid exposure induces profound, projection-specific changes in glutamatergic synapses in the NAc, notably at inputs from the PFC, basolateral amygdala (BLA), and ventral Hipp [116].

Opioid exposure and opioid withdrawal could alter both the morphology and physiological properties of MSNs in NAc. Opioid withdrawal is capable of decreasing dendritic density [137,138,139]. Meanwhile, short-term opioid withdrawal decreases the excitability of MSNs [140]; long-term opioid withdrawal is associated with an increased glutamatergic synaptic strength and intrinsic excitability of MSNs [141,142]. Moreover, it was also found that the activation of extrasynaptic NMDAR causes the inhibition of particular potassium channels, contributing to the increased intrinsic excitability of MSNs [142]. In concordance with these findings, it was also demonstrated that opioid withdrawal is associated with dendritic atrophy in D1-MSNs, but not D2-MSNs. Furthermore, it was also shown that long-term fentanyl abstinence increased the excitability and excitatory input of D1-MSNs, but fentanyl withdrawal did not alter D2-MSN excitability or excitatory input onto D2-MSNs in the NAcC [143]. In a naltrexone-precipitated withdrawal model, it was also found that the activation of D1-MSNs increased in the NAc [144]. However, it is important to note that there is also evidence that opioid withdrawal increases the activity and excitability of D2-MSNs [145]. This increase in D2-MSN excitability may happen predominantly in the NAcSh [146,147,148], or the hyperactivity of D1-MSNs and D2-MSNs occurs after different opioid abstinence durations [146]. To add another layer to the complexity of D1 and D2-MSN activation during opioid withdrawal, it is also important to note that a small (approximately 5% in the NAcC and 17% in the NAcSh) but significant subpopulation of MSNs co-expresses both D1R and D2R [101,146].

Taken together, these results suggest that opioid withdrawal is associated with the increased excitability of D1 or D2 -MSN subtypes, and the activation of MSNs is time-dependent and NAc subregion-specific.

Therapeutically, targeting glutamate signalling offers multiple entry points. NMDAR antagonists such as ketamine reduce withdrawal severity and facilitate buprenorphine induction [149], while its metabolite (2R,6R)-hydroxynorketamine reverses morphine-induced behavioural deficits and upregulates GluN2A–BDNF–linked plasticity [150]. Memantine, an NMDAR antagonist, shows preclinical efficacy in reducing morphine intake and tolerance, though clinical outcomes are mixed [151]. Restoring glutamate homeostasis via GLT-1/xCT upregulation (ceftriaxone, N-acetylcysteine) reduces [116,152]. These data suggest that, in addition to the current opioid-based treatment of OUD, non-opioid drugs of different natures have also gained the attention of researchers working in the field of SUD, including OUD. Drugs that impact the activity of NMDARs were among those investigated due to the contribution of NMDARs to the neurochemical pathology of OUD. Therefore, drugs that alter the activity of NMDARs, including their co-agonists such as D-serine or glycine, are increasingly being tested for their ability to restore psychiatric conditions such as schizophrenia and, last but not least, to delay the development of opioid analgesic tolerance and aid in recovery from SUD. The next section sheds light on future perspectives regarding the implications of GlyT-1 and Orphan G protein-coupled receptor GPR158, which functions as a metabotropic glycine receptor (mGlyR) in OUD. The contributions of the glutamatergic system to opioid disorder neuropharmacology are outlined in Table 3.

## 5. The Role of Glycine in Modulating N-Methyl-D-Aspartate Receptors: Current Insights into Opioid Addiction Therapy

The modulation of NMDAR function, particularly through the glycine site, has been extensively studied for its impact on extinction and relapse evoked by several substances of abuse. In light of the potential impact of glycine transporters regarding OUD, to the best of our knowledge, no study aimed at evaluating the therapeutic relevance of drugs affecting these transporters in OUD has been published yet. Significantly, activation of NMDAR necessitates the concurrent binding of glutamate alongside the co-agonists D-serine or glycine (via GluN1 subunits) [25,161,162]. Consequently, the inhibitors of glycine transporters, particularly GlyT-1, may prove to be a significant area of investigation of OUD. Glycine, as a neurotransmitter, also displays an excitatory character through “excitatory Gly receptors” of NMDA type, which are composed of GluN1 and GluN3A subunits and have been found in areas mediating aversive behaviours [163]. Finally, the recently identified GPR158 functions as a mGlyR, providing recent insights into the intricate nature of glycinergic transmission in the CNS [164].

The activation of NMDARs as a consequence of the elevation of glutamate in the NAc and other areas contributes to the development of OUD, as recognised by several studies that point to the ability of NMDAR antagonists, such as ketamine and MK-801, among others, to suspend the development of morphine-induced CPP in animals [165,166,167,168,169,170,171,172]. For example, morphine significantly elevates glutamate in NAc, striatum, and Hipp [153]. Other drugs, like crocin, a carotenoid from saffron (*Crocus sativus* L.), mitigate opioid withdrawal–induced glutamatergic hyperactivity and neuroinflammation, likely via downregulation of NMDA receptor subunits and attenuation of astrocytic activation, supporting its potential as an adjunctive therapy for OUD [173].

Initial investigations established that NMDAR antagonism can impair extinction. For example, the non-competitive NMDA antagonist MK-801 dose-dependently blocks the extinction of Pavlovian fear conditioning, while the intraamygdalar infusion of the competitive NMDA antagonist AP5 blocked the extinction of fear-potentiated startle [174,175]. Conversely, facilitation of extinction by the partial NMDAR agonist D-cycloserine (DCS) is also noticed in conditioned freezing [176,177] and fear-potentiated startle [178] paradigms, both after systemic and intraamygdalar administration. In an appetitive (food-reinforced) conditioning task, the NMDAR antagonist MK-801 decreases, while the agonist DCS increases responding during extinction [179]. Results are more controversial in extinction/reinstatement paradigms. DCS accelerates the extinction of cocaine-induced CPP both in rats [180,181] and mice [182]. Interestingly, knockout serotonin transporter rats show attenuation in the facilitatory effect of DCS on cocaine CPP extinction [183]. Other NMDAR co-agonists, such as sarcosine and D-serine, can also facilitate the extinction of cocaine-induced CPP, albeit with a different time course from DCS [184]. DCS also facilitates the extinction of naloxone-induced conditioned place aversion in morphine-dependent rats [185]. In opposition to the positive findings, it was reported that DCS does not affect the rate of extinction of ethanol-induced CPP in mice, though it does interfere with subsequent reconditioning [186]. The existing literature also points out that DCS has no significant effect on the extinction or reinstatement of morphine-induced CPP [187].

Glycine site and non-competitive NMDAR antagonists were found to decrease the expression of the naloxone-precipitated morphine withdrawal syndrome and to facilitate the extinction of morphine dependence [170]. The compounds also inhibited the acquisition and expression of morphine-induced CPP but did not influence its extinction.

Results are also equivocal in self-administration assays. DCS accelerated the extinction of cocaine self-administration in rats [188] and in mice [189]. An inhibition of reinstatement of both ethanol and cocaine seeking in rats that underwent extinction of ethanol/cocaine self-administration was reported for the NMDA/glycine receptor antagonist L-701,324 and the AMPA/kainate antagonist CNQX, but was not reported for the non-competitive NMDA antagonist MK-801 and the competitive antagonist CGP39551 [190,191]. In contrast, it was found that intra-basolateral-amygdala infusions of DCS potentiated the reconsolidation of cocaine-associated memories and increased cue-induced relapse to drug seeking in rats [192].

Clinical and human laboratory studies also question the extinction promoting efficacy of DCS. A trend towards increased craving to cocaine cues after DCS administration was reported [193]. This was followed by studies which concluded that cue-induced craving in cocaine-dependent individuals was not attenuated by DCS and may have even been potentiated [194,195]. This was further supported by neuroimaging data [196], which suggested that DCS may prevent the extinction of cocaine cues.

In contrast to the direct agonism at the glycine site with DCS, the manipulation of the glycine site via GlyT-1 inhibition, which increases synaptic glycine levels, has so far produced more consistent results in reducing drug-seeking behaviour. It was concluded that the GlyT-1 inhibitor RO4543338 facilitated the extinction of cocaine self-administration and attenuated subsequent reacquisition [197]. Similarly, it was found that another GlyT-1 inhibitor reduced nicotine-induced cue-potentiated reinstatement of sucrose self-administration, but did not affect food-induced reinstatement [198]. It was demonstrated that two different GlyT-1 inhibitors (SSR504734, A-1246399) reduced alcohol-seeking and relapse-like consumption, as well as abolishing cocaine-seeking responses [199]. The latter finding confirmed the results of Cervo et al. [200], who also demonstrated the effectiveness of SSR504734 in cocaine seeking, but it was also shown that cue-induced reinstatement of sucrose seeking was not affected by the compound.

Astrocytic glutamate clearance is consistently impaired in OUD. Downregulation of glial transporters GLT-1, cystine-glutamate antiporter (xCT), and modulating acid-sensing ion channel function in NAc and Hipp elevates extracellular glutamate and enhances spillover, amplifying cue-driven relapse vulnerability [45,116,201,202]. Repeated exposure to drugs of abuse, including opioids such as heroin, modifies glutamate transmission, which can subsequently induce synaptic plasticity in the NAc and VTA, ultimately contributing to the development of OUD [152].

**Figure 2:**
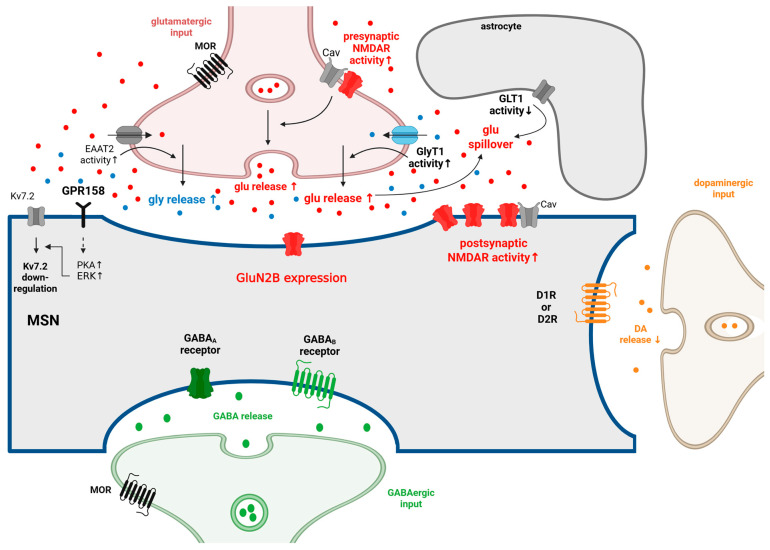
The synaptic changes at MSN in NAc during repeated and chronic opioid use and withdrawal. MSNs make synapses with DArgic neurons from the VTA. They also receive glutamatergic inputs from several brain regions, including the PFC, BLA, ventral Hipp, the paraventricular nuclei of the thalamus, and the VTA. GABAergic inputs reach the MSNs from local inhibitory interneurons and from the projecting VP neurons (for more details, see Figure 1). Several of these glutamatergic and GABAergic inputs are under the presynaptic control of MORs. MORs are expressed in VgluT2+ glutamatergic neurons, and these MORs are essential in the development of naloxone-precipitated withdrawal symptoms. During repeated opioid administration, the activity of presynaptic and postsynaptic NMDAR, as well as their coupling with calcium channels, increases in the MSNs. The concentration of extracellular glutamate could increase, especially during opioid withdrawal. The reason behind this could be that the activity of GLT1 is decreased in astrocytes during OUD, and glutamate release is increased in OUD as well. In the NAc, GluN2B-containing NMDARs play a crucial role in the development of OUD. The elevated glutamate level increases the release of glycine via glycine heterotransporters located on the glutamatergic axon terminals. Glycine is capable of modulating GPR158, which is expressed on MSNs. As a recent finding, GPR158 is a metabotropic glycine receptor that activates PKA-ERK signalling in the presence of glycine. As a result of the activation of the GPR158/PKA/ERK pathway, the expression of Kv7.2 is downregulated. The downregulation of the Kv7.2 potassium channel and the increased activity of NMDARs contribute to the hyperexcitability of MSNs, which is observed during repeated opioid administration and withdrawal. During withdrawal, DA release decreases from VTA DArgic neurons due to the increased activity of GABAergic neurons projecting to the VTA DArgic neurons. In the NAc, the GABA release is increased in acute withdrawal. In accordance with this, it was shown that the excitability of MSNs decreased during acute opioid withdrawal, and hyperexcitability of MSNs could be detected during long-term opioid withdrawal. ↑: increase; ↓: decrease. Adopted from [45,108,120,130,140,141,143,161,205,208,209,210,211,212,213,214,215,216,217,218,219]. Created in BioRender. Gergely, T. (2025) https://BioRender.com/u2oekl0.

Pharmacological GLT-1/xCT upregulation with ceftriaxone or N-acetylcysteine restores homeostasis and reduces reinstatement [116,152,203]. During morphine dependence and withdrawal, glutamate levels, astrocytic GFAP, and pro-inflammatory cytokines interleukin-1β and tumour necrosis factor-α rise across striatum, NAc, and Hipp; crocin co-treatment normalises these measures, downregulates Grin1/Grin2A genes, and reduces withdrawal scores [153]. Furthermore, a previous work has shown that glycine can elicit a concentration-dependent release of 3H D-aspartate. The enhancement of spontaneous release of this amino acid is likely mediated by the activation of a glycine transporter. Interestingly, the effects of glycine were antagonised by the glycine transporter inhibitor glycyldodecylamide, but not by strychnine, the antagonist of ligand-gated ion channel glycine receptors that mediate inhibitory neurotransmission in the CNS [204]. Previous research has highlighted the interplay between glycine and glutamate in relation to heterotransporters, indicating that the neuronal uptake of one neurotransmitter can stimulate the release of another [161,204]. In the context of OUD, where there is an elevation in glutamate levels, this dynamic may lead to an increased release of glycine. More recently, glycine-dependent activation of GPR158 was found to increase the firing rate of NAc MSNs [205]. The question is whether glycine transporter inhibitors, particularly those targeting GlyT-1, predominantly found in astrocytes, could reinstate the disrupted synaptic homeostasis associated with OUD. In this regard, future studies are required to elaborate on the impact of glycine transporters, specifically GlyT-1 or GlyT-2, in OUD development. Recent evidence indicates that exogenous D-serine can reverse the inhibitory effects of morphine on the excitability of GABAergic neurons that are mediated by NMDARs in the NAc [206]. Whether the elevation of glycine levels in NAc via GlyT-1 [207] inhibition would yield results comparable to those reported in the earlier research conducted by Wu and colleagues regarding D-serine, which is a co-agonist at NMDARs like glycine. Given that serine racemase, the enzyme that mediates the conversion of L-serine to D-serine in cells, is predominantly expressed in neurons, GlyT-1 emerges as an important target of interest. In conclusion, based on the above-mentioned literature, we hypothesise that GlyT inhibitors, specifically GlyT-1, may be beneficial in the alleviation of OUD symptoms like enforcement in the following way. Inhibiting GlyT could decrease the activity of GlyT-1 heterotransporters at glutamatergic nerve terminals in the NAc. Consequently, GlyT-initiated glutamate release from these terminals may decrease, lowering extracellular glutamate levels. Lowering the extracellular glutamate concentration initiates a decrease in NMDAR overactivation and could reduce the glutamate-driven glycine release. Glycine levels could also impact GPR158 activity on MSNs. At lower glycine levels, the activity of the GPR158-initiated PKA/ERK signalling pathway is decreased, thus leading to inhibition of GPR158-initiated Kv7.2 downregulation. Taken together, attenuated NMDA-mediated glutamatergic transmission and preserved function of Kv7.2. (Figure 2). Based on the above-mentioned literature, dysregulation of glutamate homeostasis and abnormal NMDAR activity in various brain regions, particularly the NAc, play a crucial role in OUD. Active substances capable of modulating these changes, such as ketamine, have shown therapeutic potential in treating OUD. As reviewed here, GlyT inhibitors can also influence glutamate homeostasis and NMDAR activity. Therefore, we propose that GlyT-1 inhibitors may have therapeutic effects in OUD.

## Figures and Tables

**Table 1:** Preclinical evidence regarding the effect of opioid agonists on dopamine level alterations in the nucleus accumbens.

Opioid Ligand and Route of Administration	Subject	Findings	Reference
Heroin self-administration, IV	Male Long-Evans rats	↑ DA and DOPAC levels in the NAc. Increasing the fixed ratio from 1 to 10: no DA or DOPAC elevation. Extinction: ↓ DA and DOPAC levels, drug-seeking behaviour was indicated by lever pressing. Higher heroin dose: ↑ DA and DOPAC levels, showing dose-dependent enhancement of DA activity.	[33]
Fentanyl, IV and direct infusion into the VTA or the NAc (via microdialysis probe)	Male Sprague Dawley rats	↑ DA release in rat NAc by MOR, DOR1, DOR2 activation.	[31]
Morphine, IP	Female Sprague Dawley derived rats	Monotonic ↑ in DA metabolites (DOPAC and HVA in the NAc, DOPAC in the striatum), a non-monotonic ↑ in DA in the NAc and striatum.	[37]
Morphine, IV or oxycodone, infusion Naloxone, IV, then 15 min later, either morphine or oxycodone	Male Sprague Dawley rats	Oxycodone: rapid and sustained DA concentration ↑ in the NAc with rapid rises in the frequency and amplitude of phasic DA release events. Morphine: transient spike in both DA and GABA levels. Naloxone abolished the morphine and oxycodone effects on DA level.	[35]
Morphine, IP	Male Wistar rats (Charles River)	Dose-dependent ↑ DA and DOPAC and HVA release in the NAc.	[38]
Morphine or saline, SC (days 1–6). On Days 0 and 7, Morphine, IV. Naloxone, IV on days 0 and 7.	Male C57BL/6 mice	Acute and chronic morphine: ↑ DA in the medial and lateral regions of NAc. Naloxone treatment reversed the increased tone of DA in NAc.	[34]
Morphine, IP for 7 days Naloxone, IP	Male, Sprague Dawley rats	Acute and chronic morphine: ↑ DA, DOPAC, and HVA levels in NAc. Chronic morphine did not change basal DA release. Naloxone (8th day): induced withdrawal symptoms and ↓ NAc DA. These effects were reversed by pretreatment with clonidine.	[39]
Morphine, SC	Male Sprague Dawley rats	Chronic morphine: long-lasting sensitization of DA release in the NAc. Challenge dose (either 3 days or 30 days later): ↑ DA release in NAc.	[36]
fentanyl (SC 20 or 30 μg/kg), (IV 10 μg/kg), or self-administered by rats (2.5 μg/kg/infusion)	Male adult Wistar rats	SC and IV Fentanyl: induced CPP and ↑ DA release in NAcSh, which was reversed by pretreatment with the GHS-R1A antagonist, JMV2959 (IP, 1 or 3 mg/kg) given 20 min before three consequents daily 360 min IV self-administration sessions under a fixed ratio. JMV2959 affected the concentration of byproducts associated with DA metabolism in the NAc (↑ DOPAC and HVA levels after s.c. fentanyl injection, accelerated early HVA formation following IV fentanyl, and ↓ the peak HVA levels (20%), with no impact on 3-MT).	[42]
Morphine, SC, and last injection IP. Naloxone, SC	Male rats (Wistar-Kioto, Charles River)	One day after morphine withdrawal, dependent rats showed lower DA levels in the caudal NAc-ventral striatum. A morphine challenge dose: ↑ DA in controls but had no effect in withdrawn animals.	[40]
Oxycodone, intermittent access. Next, a period of forced abstinence was imposed.	Adult female and male Long Evans rats	Oxycodone abstinence: ↓ NAcC DA uptake. Oxycodone ↓ DA uptake in abstinent rats. ↓ in DA transporter phosphorylation, only on day 15 of abstinence. Pretreatment with naloxone or CTAP significantly attenuated the effects of oxycodone on DA uptake.	[41]

CTAP: (D-Phe-Cys-Tyr-D-Trp-Arg-Thr-Pen-Thr-NH2); IV: intravenous; IP: intraperitoneal; SC: subcutaneous; ↑: increase; ↓: decrease.

**Table 3:** Contributions of the glutamatergic system to opioid use disorder neuropharmacology.

Opioid Ligand and Route of Administration	Subject	Findings	Reference
DAMGO, DPDPE, U69593 (brain slice electrophysiology)	Male and female C57Bl/6J mice	ORs differentially regulate cortical glutamate circuits in the CLA–ACC. MOR/DOR activation modulated postsynaptic recurrent excitation, whereas presynaptic KOR activation inhibited glutamate release.	[112]
Morphine, IP (acute and chronic)	Male Wistar rats	Morphine induces widespread neuroinflammation and upregulates glutamatergic signalling through upregulation of glutamate, GluN3B NMDAR mRNAs, and astrocytic/inflammatory markers in different brain areas (NAc, striatum, Hipp).	[153]
Morphine, SC Hydroxynorketamine, IP Naloxone, IP	Male and female C57BL/6J mice	The metabolite hydroxynorketamine facilitates extinction, reverses morphine CPP, and prevents relapse by restoring NMDAR function and cortical EEG, increasing GluN2A and BDNF levels (GluN2A-dependent) in ventral Hipp and PFC.	[150]
Oxycodone self-administration, IV	Male Sprague Dawley rats	Glutamatergic projections from OFC to DS, coupled with D1R signalling, drive incubated oxycodone craving, whereas circuit disconnection reduces drug-seeking behaviour.	[118]
Heroin self-administration, IV	Male Sprague Dawley rats	Heroin suppressed VPGlu neuron activity during self-administration and relapse, while extinction reversed this pathway. Extinction increased excitatory (Vglut2) and decreased inhibitory (Vgat) markers in LHb terminals. Activation of VPGlu or its projections to the LHb suppressed heroin seeking, indicating that enhanced VPGlu–LHb glutamatergic transmission after extinction suppressed seeking.	[119]
DAMGO, CTOP (bath application)	Adult male Kunming mice (cerebellum slices)	Presynaptic MOR activation inhibited glutamatergic transmission at parallel fibre–Purkinje cell (PF–PC) synapses by reducing presynaptic glutamate release via cAMP–PKA and MAPK–ERK signalling.	[123]
Cocaine, morphine, nicotine, IP	Wild-type and transgenic mice (C57/BL6, DATKI and DAT-Cre mice)	Addictive drugs induce redistribution and insertion of GluA2-lacking calcium-permeable AMPARs in VTA DA neurons. This effect depends on DA release patterns that drive D1R-dependent synaptic plasticity.	[115]
Oxycodone, cocaine, IV	Male young adult Long Evans rats	Polydrug use downregulated GLT-1, altered AMPA/NMDA ratio, and exacerbated glutamate dyshomeostasis in NAcC.	[120]
Oxycodone self-administration, IV	Male Sprague Dawley rats	Oxycodone caused incubation of craving after long withdrawal and induced specific changes in hippocampal glutamate receptor expression linked to craving (upregulated ionotropic, downregulated mGluR2/3, upregulated other mGluR subunit mRNAs).	[117]
Heroin self-administration, IV	Male Long Evans rats	CRF1 upregulation in VTA DA neurons and NAc co-expressing GluR1 promotes opioid use.	[154]
Oxycodone, IP	Male and female adult Sprague Dawley rats	Glutamatergic adaptations to opioids are highly influenced by sex and stress history. Females downregulated NMDA after CPP; stressed males increased postsynaptic glutamate receptors within the hippocampal CA3.	[121]
Morphine, SC	Male ICR mice	Enhanced AMPAR signalling in descending pain inhibitory circuits (PAG–RVM pathway) prevented morphine tolerance and dependence.	[155]
Leu-enkephalin, DAMGO, DPDPE, morphine, (_)-U-50,488	Male CD-1 mice (mPFC slices)	Opioid and DR signalling synergistically enhance cortical excitability. OR βγ subunits potentiate D1R-stimulated adenylate cyclase, enhancing AMPAR/NMDAR phosphorylation in the mPFC, which contributes to opioid addiction regulation.	[156]
Anatomical and immunocytochemical investigation	Male C57/BL/6 mice	GluR2-containing AMPARs co-localise with MOR in CeA dendrites, supporting direct opioid modulation of amygdala excitability.	[157]
Morphine, SC Dextromethorphan, IP	Male Sprague Dawley rats	Dextromethorphan attenuates morphine reward via activation of S1Rs in the VTA.	[158]
DAMGO, CTAP, met-enkephalin, morphine sulphate (mHb–IPN brain slices)	Male and female transgenic mice	MORs differentially modulate distinct neurotransmitter pathways in the mHb–IPN. MORs potentiate cholinergic → IPR glutamate transmission while Kv1 channel blockade unmasked MOR-enhanced nAChR-mediated EPSCs, revealing opioid control of excitatory signalling.	[159]
Oxycodone self-administration, oral	Male and female C57BL/6J and VGluT2-IRES::Cre mice	MORs inhibit glutamatergic input to VTA DA neurons, and VGluT2+ neurons projecting to the NAc are active during seeking. Inhibiting these neurons reduces reinstatement in males, only indicating a sex-dependent effect.	[113]
Heroin self-administration, IV in rats	Male Long-Evans rats (Translational); human postmortem	In OFC, Shisa7 is downregulated after heroin; its overexpression increases heroin seeking and impairs cognitive flexibility by altering glutamate/GABA receptor interactions.	[132]
OUD (opioid ligand was not specified)	Human postmortem	OUD disrupts circadian regulation of glutamatergic/GABAergic genes in DLPFC and NAc, potentially contributing to sleep and relapse issues.	[135]
Systematic review	Humans with OUD and opioid withdrawal	Adjunctive high-dose ketamine shows promise for treating OUD and withdrawal by targeting the glutamatergic system.	[149]
Systematic review	Preclinical (animals) and clinical trials (humans)	Memantine decreases OUD behaviours (craving, consumption, and withdrawal) severity in animal models, but in humans, the results were mixed but positive in terms of cognitive function. Memantine reduced morphine self-administration, CPP, tolerance, and withdrawal in animals through NMDAR antagonism in different brain areas.	[151]
Heroin (abused for over 1 year)	Human (males)	Longer promoter repeats in GRIN2A L-allele carriers reduced GRIN2A transcription, altering NMDA function, causing higher heroin craving.	[160]

## Data Availability

No new data were created or analyzed in this study. Data sharing is not applicable to this article.

## Abbreviations

The following abbreviations are used in this manuscript:

NSOs	Novel synthetic opioids
IV	Intravenous
Fentalogs	Fentanyl analogues
MORs	µ-Opioid receptors
OUD	Opioid use disorder
SUD	Substance use disorders
LH	Lateral hypothalamus
MH	Medial hypothalamus
PAG	Periaqueductal Gray
DRF	Dorsal part of the reticular formation
VRF	Ventral part of the reticular formation
VTA	Ventral tegmental area
NAc	Nucleus accumbens
CPP	Conditioned place preference
SN	Substantia nigra
non-ORs	Non-opioid receptors
MOUD	Medications for opioid use disorder
DA	Dopamine
NMDA	N-Nethyl-D-aspartate
GABA	Gamma-aminobutyric acid
NMDARs	N-Methyl-D-aspartate receptors
Hipp	Hippocampus
NAcSh	NAc shell
Glu	Glutamate
AMPA	α-Amino-3-hydroxy-5-methyl-4-isoxazolepropionic acid
NAcC	NAc core
GlyT-1	Glycine transporter 1
DOR2	δ_2_-Opioid receptor
DOPAC	3,4-Dihydroxyphenilacetic acid
HVA	Homovanillic acid
DAMGO	D-Ala2,N-Me-Phe4,Gly5-ol-enkephalin
DAT	DA transporter
CTAP	D-Phe-Cys-Tyr-D-Trp-Arg-Thr-Pen-Thr-NH2
MSN	Medium spiny neuron
D1-MSNs	D1 receptors
D2-MSNs	D2 receptors
RMTg	Rostromedial tegmental nucleus
IPSCs	Inhibitory postsynaptic currents
GVG	γ-Vinyl GABA
CeA	The central amygdala
PKA	Protein Kinase A
BDNF	Brain-derived neurotrophic factor
CREB	Cyclic AMP response element-binding protein
MoCA	Montreal Cognitive Assessment
PFC	Prefrontal cortex
SNP	Single-nucleotide polymorphism
mNAcSh	Medial nucleus accumbens shell
DR	Dorsal raphe
VGaT	Vesicular GABA transporter
VGluT3	Vesicular glutamate transporter 3
TPH	Tryptophan hydroxylase
aMCC	The anterior midcingulate cortex
GAD	Glutamate decarboxylase
AIC	The anterior insular cortex
GAD-ir	GAD-immunoreactive
PAMs	Positive allosteric modulators
GIRK	G-protein-gated inwardly rectifying potassium channel
RGS2	Regulator of G protein signalling 2
CNS	Central nervous system
PV	Parvalbumin
SST	Somatostatin
VIP	Vasoactive intestinal peptide
O-LM	Oriens-lacunosum moleculare interneurons
NRM	Nucleus raphe magnus
PLA2	Phospholipase A(2)
PKC	Protein kinase C
N/OFQ	Nociceptin/orphanin FQ peptide
CLA–ACC	Claustrum–anterior cingulate cortex
KOR	κ-Opioid receptor
EPSCs	Excitatory post-synaptic currents
CINs	Cholinergic interneurons
D1R	D1 receptors
D2R	D2 receptors
BLA	Basolateral amygdala
VP	Ventral pallidum
LDT	Lateral dorsomedial tegmentum
AMPARs	AMPA receptors
GLT-1	Glutamate transporter-1
CIS	Chronic immobilisation stress
OFC	Orbitofrontal cortex
DS	Dorsal striatum
LHb	Lateral habenula
VPGlu	Glutamatergic neurons in the ventral pallidum
PF–PC	Parallel fibre–Purkinje cell
ERK	Extracellular signal-regulated kinases
LTP	Long-term potentiation
pGLUN1	Phosphorylation of Ser897 in the GLUN1 subunit
DLPFC	The dorsolateral prefrontal cortex
mGlyR	Metabotropic glycine receptor
GFAP	Glial fibrillary acidic protein
HNK	Hydroxynorketamine
CRF1	Corticotropin-releasing factor
RVM	Rostral ventromedial medulla
mHb–IPN	The medial habenula–interpeduncular nucleus
Kv1	K^+^-channels of the shaker family
nAchR	Nicotinic acetylcholine receptor
xCT	Cystine/glutamate antiporter
S1R	Sigma-1 receptor
DCS	D-Cycloserine
